# Is Intravascular Lithotripsy (IVL) Alone a Therapy for the Treatment of Aortoiliac Occlusive Disease (AIOD)?

**DOI:** 10.7759/cureus.47537

**Published:** 2023-10-23

**Authors:** Rutaba Azim, Daniel I Razick, Fayez S Siddiqui, Rafael Landeros, Sajid Ali

**Affiliations:** 1 Cardiology, Houston Cardiology Consultants, Houston, USA; 2 Surgery, California Northstate University College of Medicine, Elk Grove, USA; 3 Internal Medicine, California Northstate University, Elk Grove, USA

**Keywords:** peripheral vascular diseases, interventional cardiology, shock wave lithotripsy, bilateral iliac artery occlusion, atherosclerosis

## Abstract

We present a rare case in which a 63-year-old male with a history of hypertension, diabetes mellitus, hyperlipidemia, and previous coronary artery bypass graft (CABG) presented with bilateral external iliac artery near occlusion. We describe the utilization of lithotripsy balloon angioplasty as opposed to the traditional double-barrel stenting method or modified endovascular repair (EVAR) to treat the occlusion. Pre-operative computed tomography (CT) angiography demonstrated a 90 percent occlusion of both the distal aorta and right external iliac artery, and 99 percent occlusion of the left external iliac. The patient remains symptom-free three years post-intervention with normal right and left ankle-brachial indices, 1.34 and 1.32 respectively. We review the available literature regarding aortoiliac occlusive disease (AIOD) and discuss the advantages and disadvantages of novel and traditional treatment modalities. Understanding all treatment options is crucial for physicians who are presented with similar cases.

## Introduction

The descending aorta bifurcates and gives rise to the common iliac arteries, which bifurcates once more to give rise to the external and internal iliac arteries. The larger external iliac artery forms the main blood supply to the lower extremity, while the internal iliac artery primarily supplies reproductive organs, the pelvis, pelvic organs, and the medial thigh. Any change in diameter or disease at the iliac arteries plays an important role in planning an endovascular intervention due to the possible complications that a blood flow restriction may produce [1].

Many patients with aortoiliac occlusive disease (AIOD) and peripheral artery disease are asymptomatic. While this makes determining the exact prevalence of the diseases in the general population difficult, estimates range from 3.56 percent to greater than 14%. Presenting severity and onset dictate the management and treatment of AIOD [2]. 

There are a few techniques used to repair iliac artery and aorta lesions, one of them being angioplasty. Angioplasty, performed with or without stenting, is a percutaneous procedure used to widen occluded arteries due to underlying atherosclerosis [3]. The procedure is done by navigating a balloon-tipped catheter to the stenosed site and inflating the balloon to widen the luminal diameter and normalize blood flow [3]. Plain old balloon angioplasty (POBA) was the first step on the path of modern coronary intervention with positive results and areas of opportunity for future methods that subsequently appeared, such as stenting [4].

Currently, double-barrel stenting or modified endovascular aneurysm repair has been commonly utilized in the treatment of visceral artery dissecting aneurysms and bilateral iliac artery aneurysms [5,6]. A commonly encountered complication is the compression of one stent by the other, causing a new outflow stenosis making future access more difficult [7]. To avoid this possible complication, consideration for lithotripsy balloon angioplasty alone can be made.

We present a rare case in which the use of lithotripsy balloon angioplasty alone in a patient with bilateral iliac near occlusion with severe distal aortic closure was performed, opposing the conventional stenting approach. Traditional successful patency outcomes due to open repair have been replaced with endovascular stenting for short proximal lesions. In the setting of aortoiliac obstruction, it is, however, not the only treatment modality, and balloon angioplasty with lithotripsy was considered due to clinical expertise and the surgeon’s comfort.

## Case presentation

**Figure 1:**
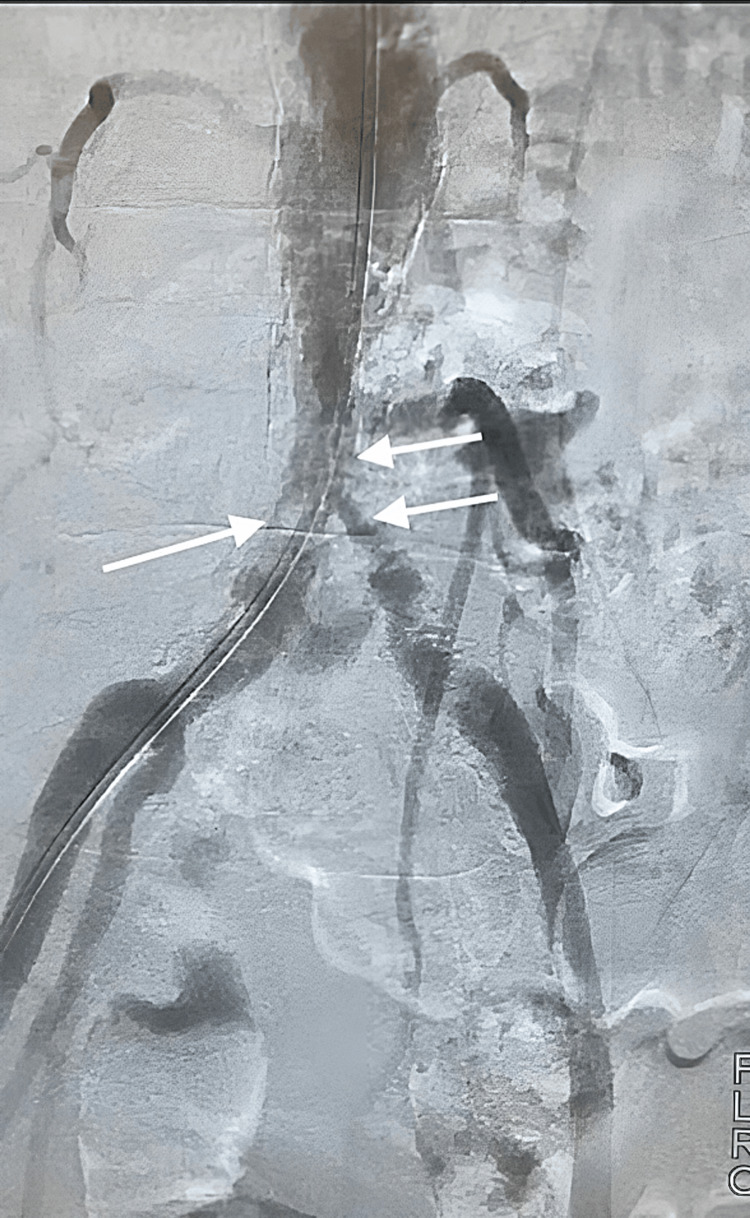
Pre-operative computed tomography (CT) angiography displaying severely calcified arteries with moderate stenosis of distal aorta and severe stenosis of bilateral common iliac arteries.

A 63-year-old male with a history of hypertension, hyperlipidemia, DM, 35-pack year smoking history, and coronary artery disease (CAD) status post coronary artery bypass graft (CABG) presented to an outpatient clinic for bilateral leg pain during exertion and at rest. Physical exam revealed absent right popliteal pulse and bilateral hypoesthesia of lower shins and feet. Chronic limb ischemia was suspected and Doppler ultrasound was used to measure pulse volume waveform at segmental locations in the leg arteries. To confirm the diagnosis further, computed tomography (CT) angiography was performed revealing a 90% distal aorta, 99% left external iliac artery, and 90% right external iliac artery stenosis (Figure 1). The patient had refractory symptoms despite graded exercise therapy, and medical therapy with the decision to proceed with possible angioplasty, modified endovascular repair (EVAR) was made.

**Figure 2:**
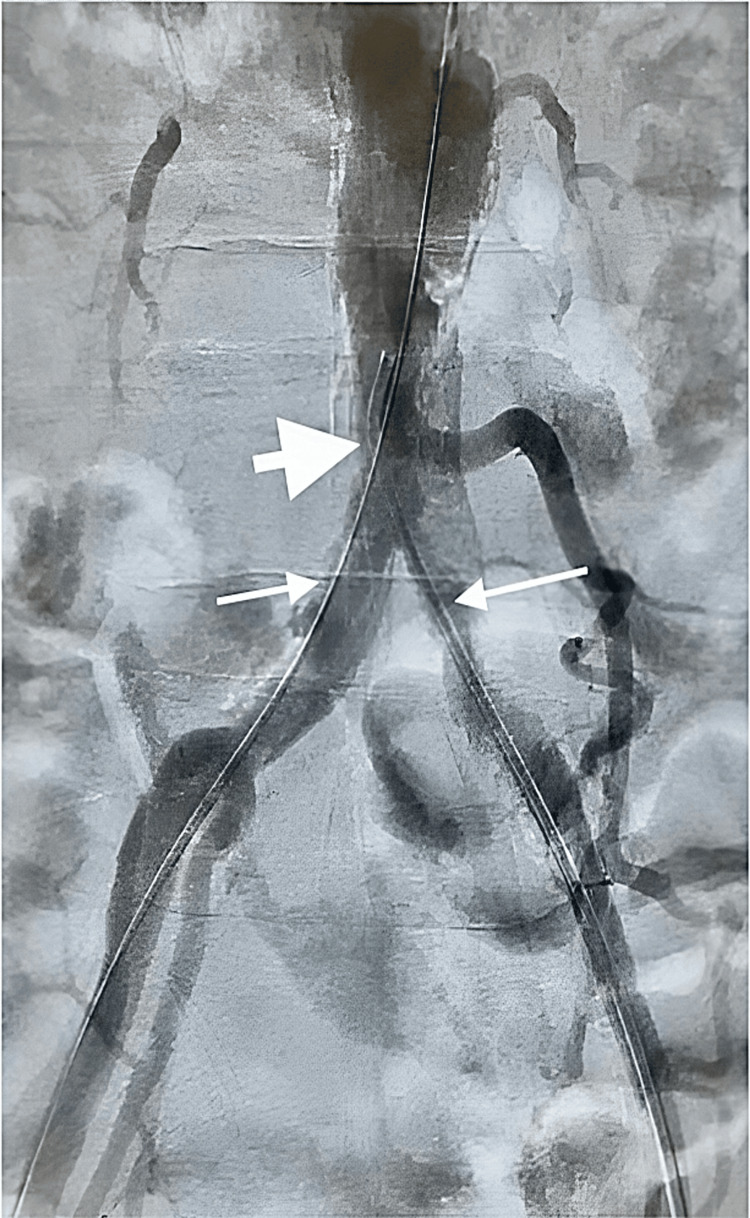
Post lithotripsy balloon angioplasty computed tomography (CT) angiography demonstrating marked improvement in distal aorta and bilateral external iliac artery blood flow.

No contraindications for surgery were present and due to the diagnosis of AIOD and the multilevel nature of occlusion, operative intervention was planned with the goal of revascularization. After obtaining bilateral femoral access, a 0.35 glidewire advantage micro-catheter was used to cross the lesions. Angiogram images were obtained to visualize the lesions. 8.0 lithotripsy balloon was used for three cycles each at distal aorta to external iliac arteries, at 4 atm of pressure. Then bilateral kissing 8 mm balloons were used at 8 atm of pressure used for after. CT angiography demonstrated significantly improved flow with some residual stenosis in the left iliac artery (Figure 2). However, the stenosis did not limit blood flow and no flow-limiting dissections were seen. Further pressure gradients were demonstrated to be 0. 

Three years post-operation, the patient remains symptom-free with the most recent Doppler ultrasound demonstrating bi/triphasic flow in lower extremities with a normal ankle-brachial index (ABI) of 1.34 on the right and 1.32 on the left. The patient remains on clopidogrel and aspirin dual therapy and is symptom-free. 

## Discussion

In patients with severe iliac artery stenosis involving the distal aorta, double-barrel stenting or modified utilization of endovascular aortic aneurysm repair devices have been used traditionally as a percutaneous approach for intervention. However, in this case, we describe the utilization of lithotripsy balloon angioplasty at low pressure to take severe calcified stenosis to mild to moderate stenosis with no pressure gradient after. Further three years post-intervention, the patient remains symptom-free with essentially normal ABI. Understanding the advantages and disadvantages of both options is crucial for physicians who are presented with similar cases specifically when the distal aorta is involved.

Double-barrel stenting is a technique in which stents with a common origin and different outflow targets are deployed in parallel. Long-term complications can be noted if intravascular ultrasound is not performed. Further limitations with interventions arise as a new iliac bifurcation is formed with balloon expandable stents which can be crushed and create new ostial stenosis [8]. To avoid this possible complication, consideration for lithotripsy balloon angioplasty alone can be made.

Percutaneous old balloon angioplasty (POBA) is a technique that has been used since 1963 to treat arterial occlusions, in particular obstructive coronary artery disease and atherosclerosis. POBA is generally considered to be safe and quick, and patients are reported to have faster recovery times [5,6]. The percutaneous method involves the use of a catheter and a small balloon which is guided to the occlusion and inflated thus widening the diameter of the arterial lumen and restoring blood flow. However, balloon angioplasty alone has been characterized as a temporary fix, with restenosis occurring in 20-40% of patients due to elastic recoil [5]. Balloon-induced dissection is another complication in POBA that can cause abrupt vessel closure. Other potential risks include plaque extrusion and subsequent thromboemboli formation, pulmonary embolism, or stroke [6].

Lithotripsy balloons have been recently introduced to address and minimize these risks specifically with calcified vessels utilized at very low pressures. Intravascular lithotripsy (IVL) consists of a balloon-based system with lithotripsy emitters that create cracks in calcified or atherosclerotic obstructions [9]. Data has shown that IVL leads to greater vessel compliance and has an excellent safety profile [10]. In this case, IVL was utilized and three years later, the patient has no vascular complaints or symptoms and continues to present with normal ankle brachial indices, without the need for subsequent intervention. This case demonstrates that if a patient presents with bilateral iliac artery occlusions, the use of double-barrel stenting should be re-evaluated and potentially replaced with IVL if deemed appropriate. One consideration is to start with lithotripsy alone at low pressures and if results show no significant anterograde dissections on intravascular ultrasound with resolution in pressure gradient one may consider antiplatelet therapy before placing stents. In our case, we were considering modified EVAR for the patient however once the results of lithotripsy POBA alone were seen we decided to evaluate the patient prospectively. To our surprise, he has been leg pain-free with stable ABI for three years. 

## Conclusions

While traditional approaches to bilateral iliac artery stenosis have reported favorable short-term outcomes, continued improvement in the long-term through the use of novel techniques such as lithotripsy balloon angioplasty has the potential to mold the way patients are treated for years to come. The patient discussed was a candidate for several treatment options, including double-barrel stenting and intravascular lithotripsy (IVL) balloon angioplasty. Given the heightened risk of long-term complications and based on the surgeon's comfort, the decision was made to perform IVL angioplasty. Three years later, the patient is symptom-free and shows no signs of restenosis while on continued medical management. This case highlights the value in analyzing all treatment options and selecting which is most suitable for the individual patient, as opposed to utilizing conventional approaches based on history, specifically beneficial in distal aorta-iliac pre-ostial stenosis.

## References

[1] Hammond E, Nassereddin A, Costanza M (2023). Anatomy, abdomen and pelvis: external iliac arteries. StatPearls [Internet].

[2] Heaton J, Khan YS (2023). Aortoiliac occlusive disease. StatPearls [Internet].

[3] Chhabra L, Zain MA, Siddiqui WJ (2023). Angioplasty. StatPearls [Internet].

[4] McKavanagh P, Zawadowski G, Ahmed N, Kutryk M (2018). The evolution of coronary stents. Expert Rev Cardiovasc Ther.

[5] Panchavinnin P, Tresukosol D, Phankingthongkum R, Sriyuthasak O, Sahasakul Y, Thongsawas R (2004). Stent placement compared with balloon angioplasty for obstructed coronary artery disease in Thai elderly patients: initial result and 6 months follow-up. J Med Assoc Thai.

[6] Tomberli B, Mattesini A, Baldereschi GI, Di Mario C (2018). A brief history of coronary artery stents. Rev Esp Cardiol (Engl Ed).

[7] Barton M, Grüntzig J, Husmann M, Rösch J (2014). Balloon angioplasty - the legacy of Andreas Grüntzig, M.D. (1939-1985). Front Cardiovasc Med.

[8] Ilonzo N, George JM, Price L, McKinsey JF (2021). Double-barrel stenting for endovascular repair of a superior mesenteric artery dissecting aneurysm. J Vasc Surg Cases Innov Tech.

[9] Ho HH, Lee JH, Khoo DZ, Hpone KK, Li KF (2021). Shockwave intravascular lithotripsy and drug-coated balloon angioplasty in calcified coronary arteries: preliminary experience in two cases. J Geriatr Cardiol.

[10] Honton B, Monsegu J (2022). Best practice in intravascular lithotripsy. Interv Cardiol.

